# Structural Basis for the Specific Neutralization of Stx2a with a Camelid Single Domain Antibody Fragment

**DOI:** 10.3390/toxins10030108

**Published:** 2018-03-01

**Authors:** Robert Alvin Bernedo-Navarro, Ema Romão, Tomomasa Yano, Joar Pinto, Henri De Greve, Yann G.-J. Sterckx, Serge Muyldermans

**Affiliations:** 1 Laboratory of Bacterial Genetics, Institute of Biology, University of Campinas (UNICAMP), São Paulo 13083-862, Brazil; alvinbn@gmail.com (R.A.B.-N.); tyano@unicamp.br (T.Y.); 2 Cellular and Molecular Immunology, Vrije Universiteit Brussel, 1050 Brussels, Belgium; ema.estevens.romao@vub.be (E.R.); joar.pinto@vub.be (J.P.); yann.sterckx@vub.be (Y.G.-J.S.); 3 Structural Molecular Microbiology, Vlaams Instituut voor Biotechnologie (VIB), 1050 Brussels, Belgium; henri.de.greve@vub.be; 4 Structural Biology Brussels, Vrije Universiteit Brussel, 1050 Brussels, Belgium

**Keywords:** nanobody, Shiga toxin, Stx2, B domain, neutralization, crystal structure

## Abstract

Background: Shiga toxin-producing *Escherichia coli* (STEC) are a subset of pathogens leading to illnesses such as diarrhea, hemolytic uremic syndrome and even death. The Shiga toxins are the main virulence factors and divided in two groups: Stx1 and Stx2, of which the latter is more frequently associated with severe pathologies in humans. Results: An immune library of nanobodies (Nbs) was constructed after immunizing an alpaca with recombinant Shiga toxin-2a B subunit (rStx2aB), to retrieve multiple rStx2aB-specific Nbs. The specificity of five Nbs towards rStx2aB was confirmed in ELISA and Western blot. Nb113 had the highest affinity (9.6 nM) and its bivalent construct exhibited a 100-fold higher functional affinity. The structure of the Nb113 in complex with rStx2aB was determined via X-ray crystallography. The crystal structure of the Nb113–rStx2aB complex revealed that five copies of Nb113 bind to the rStx2aB pentamer and that the Nb113 epitope overlaps with the Gb3 binding site, thereby providing a structural basis for the neutralization of Stx2a by Nb113 that was observed on Vero cells. Finally, the tandem-repeated, bivalent Nb113_2_ exhibits a higher toxin neutralization capacity compared to monovalent Nb113. Conclusions: The Nb of highest affinity for rStx2aB is also the best Stx2a and Stx2c toxin neutralizing Nb, especially in a bivalent format. This lead Nb neutralizes Stx2a by competing for the Gb3 receptor. The fusion of the bivalent Nb113_2_ with a serum albumin specific Nb is expected to combine high toxin neutralization potential with prolonged blood circulation.

## 1. Introduction

Shiga toxin-producing *Escherichia coli* (STEC) is a heterogeneous group of microorganisms, which causes around three million cases of (potentially fatal) acute illnesses in humans each year [1]. A subset of STEC, the enterohemorrhagic *E. coli* (EHEC) pathotype, is comprised of strains that are typically associated with illnesses in humans. Besides asymptomatic cases, EHEC infections lead to clinical manifestations that are fatal in some cases. The symptoms range from acute watery diarrhea and hemorrhagic colitis to hemolytic uremic syndrome (HUS), a life-threatening renal dysfunction developing approximately one week after onset of diarrhea [2]. The average cost per STEC case varies greatly according to the severity of the illness in patients. It ranges from less than $30 for those that do not require medical care to more than $6 million for patients with HUS with eventually a fatal outcome [3,4]. 

The ability of STEC strains to cause severe disease in humans is mainly related to their capacity to produce potent cytotoxins called Shiga toxins (Stx), which bear structural and functional similarity with the toxin produced by *Shigella dysenteriae* type 1 [5]. The STEC strains may produce Shiga toxin 1 (Stx1) and/or Shiga toxin 2 (Stx2) or one of their variants [5]. Several epidemiological reports suggest that HUS is more frequently associated with infections by strains producing Stx2 alone or in combination with Stx1, rather than those producing Stx1 alone [6,7,8]. In 2011, one of the largest STEC outbreaks of severe disease, linked to the consumption of fenugreek sprouts contaminated with a novel “hybrid” Stx2a-producing entero-aggregative *E. coli* (EAEC) serotype O104:H4, occurred in Germany and spread to several countries from central Europe [9]. This “deadly combination” of an already virulent strain, coupled to the acquisition of the Stx2a phage by horizontal gene transfer, caused more than 900 cases of HUS, leading to 54 deaths and large economic losses [10]. This outbreak revealed a lack of specific HUS treatments and of an effective and specific anti-Stx2 therapy [11].

The Shiga toxins belong to the ribosome-inactivating proteins (RIPs) [12] and are part of the AB5 family of toxins, which consist of an enzymatically active A subunit and a nontoxic B moiety responsible for binding to cellular receptors. The Stx B subunit comprises five identical copies of the B domain (~7.7 kDa per domain) arranged in a ring-shaped pentamer with a central pore in which the C-terminus of the monomeric A subunit is anchored [13,14]. The cascade of molecular events leading to Stx-mediated damage of host cells is well established. Through its B5 domain, the Stx holotoxin binds to the globotriaosylceramide (Gb3) receptor present on the surface of target cells, leading to subsequent internalization of the toxin [15]. The interaction between Stx and Gb3 receptor leads to uptake of the toxin/receptor complex mainly through a clathrin-dependent process [16]. After internalization, the A subunit is cleaved into the enzymatically active A1 fragment and a smaller A2 fragment [17]. The A1 fragment modifies the ribosomal RNA irreversibly through its N-glycosidase activity, leading to an arrest of protein synthesis followed by cellular death and apoptosis. These initial events lead to the first clinical manifestations of HUS. The Stx-mediated damage triggers a cascade of events that lead to the formation of thrombi in the kidney and may affect also the central nervous system, followed by long-term clinical manifestations such as severe renal disease [18,19,20].

A thorough understanding of the molecular events leading to pathology have certainly improved the prospects of a successful disease treatment [10]. The majority of therapeutic agents for the treatment of STEC infections and HUS are categorized into compounds: (i) targeting the bacteria without increasing Stx synthesis; (ii) inhibiting B5 domain-mediated binding of Stx to its receptor cells; (iii) interfering with the steps after Stx internalization by the host cell; and (iv) treating HUS sequels [21,22]. This suggests that the initial recognition event between Stx and the Gb3 receptor is a critical step for Stx-mediated damage of host cells and may be an interesting avenue for the development of novel therapeutics. Indeed, small molecules targeting the Gb3 binding site on the Stx B5 domain have been shown to neutralize the Stx activity [23]. Moreover, several reports suggest that the higher toxicity of Stx2 is due to its B subunits. The comparison in toxicity between wild-type and chimeric Stx in animal models showed that the presence of the Stx2 B subunit is critical for lethality in vivo [24,25,26]. Interestingly, in absence of the Stx A subunit, the Stx B pentamer adopts a structure that is functionally equivalent to the complete holotoxin for binding to Gb3 receptor [27] and cell internalization [16]. 

The last years have witnessed an increasing interest in the use of camelid-derived VHH single domain antibody fragments (nanobodies, Nbs) for the identification of Stx neutralizers and/or for the development of potential therapeutics for HUS treatment [28,29,30]. The study conducted by Lo et al. [30] reports the identification and structural characterization of a neutralizing Nb against the B5 domain of the Stx2e variant associated with edema disease in pigs. The articles of Tremblay et al. [28] and Mejias et al. [29] describe the identification of inhibitory Nbs against Stx variants associated with human clinical cases. While both studies present promising results on the potential use of the anti-Stx Nbs in a therapeutic setting, they lack a structural basis for Nb-mediated Stx neutralization.

In this study, we describe the identification and characterization of a nanobody (Nb113) with the potential to neutralize the Stx2a and Stx2c toxins that are associated with human clinical infections [6,7,8]. The structural basis for Nb113-mediated neutralization of Stx2a toxicity has been revealed by determining the crystal structure of the complex between Nb113 and the Stx2a B5 domain. Each B subunit in the pentameric B5 ring is associated with a single Nb113 molecule. A detailed analysis of the epitope targeted by Nb113 suggests that this Nb prevents the formation of the Stx2a–Gb3 complex, thereby impeding the subsequent steps of the internalization and enzymatic activity of the Stx2a holotoxin.

## 2. Results

### 2.1. Production of Recombinant Stx2a B Domain and Construction of an Immune Nb Phage Display Library

It is well established that the Stx B5 domain, even in absence of the Stx A subunit, forms a pentameric architecture that is functionally equivalent to the Stx holotoxin in terms of Gb3 receptor binding [27]. Therefore, the B domain of Stx2a was selected as the target for retrieving antigen-specific Nbs from an immune library. A recombinant version of Stx2a B domain (rStx2aB) was produced in *E. coli* BL21 and successfully purified by immobilized metal affinity chromatography (IMAC) and size exclusion chromatography (SEC) (Supplementary Materials, Figure S1). The rStx2aB, eluting from SEC as a pentavalent complex, was used as an immunogen to raise an immune response in the heavy chain only antibodies, specific of camelids [31]. The antigen-binding fragments from the repertoire of heavy chain-only antibodies from peripheral blood lymphocytes were cloned in our pMECS phage display vector [32] and we obtained a library size of 3 × 10^8^ individual transformants. Approximately 75% of the transformants contained an insert in the pMECS vector with the expected size of a VHH, as inferred from the PCR fragment length of 48 colonies chosen at random from the library. Forty-seven clones obtained after both, the second and third round of panning on rStx2aB, immobilized in wells of microtiter plates, were picked at random and cultured. A periplasmic extract of these bacteria was taken and used in an ELISA. Out of these ninety-four, sixty-seven clones expressed a Nb that recognized rStx2aB. The plasmid DNA of these clones was purified and the insert was sequenced. The in silico amino acid sequence analysis revealed that these nanobodies can be categorized in four families, based on differences in their third complementarity-determining region (CDR3). It has been documented that nanobodies from the same family share a CDR3 of highly similar length and sequence. These nanobodies belonging to a single family are derived from the same B-cell lineage and will target an identical epitope [33].

### 2.2. Selection of rStx2aB-Binding Nanobodies

A total of five different nanobodies binding to the rStx2aB protein were selected (referred to as Nb29, Nb31, Nb41, Nb113 and Nb140). Most of these nanobodies belong to different families since they have highly diversified CDR3s. However, Nb41 and Nb140 belong to the same family as their CDR3 differs in only two amino acids. Only Nb29 harbors the framework region 2 (FR2) amino acids characteristic for a VHH (Phe42, Glu49, Arg50 and Gly52), while all the other nanobodies have a VH imprint in FR2 (Val42, Gly49, Pro50 and Trp52) [33,34]. The molecular mass, isoelectric point (pI) and extinction coefficient for all nanobodies were calculated in silico using the ExPasy ProtParam online platform (Supplementary Materials, Table S1).

**Figure 1:**
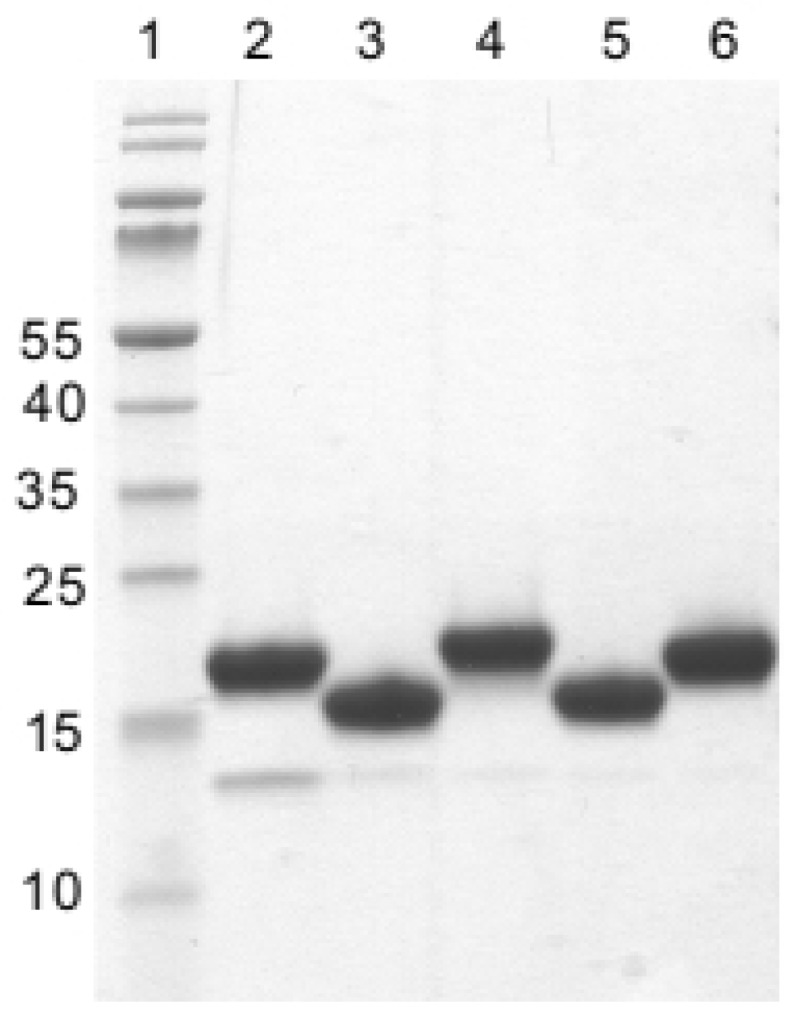
SDS-PAGE of purified rStx2aB-specific nanobodies after staining with Coomassie blue. Lane 1: protein size marker with molecular mass in kDa indicated (left); Lanes 2 to 6: Nb29, Nb31, Nb41, Nb113 and Nb140, respectively.

The pMECS plasmids containing these Nb genes were purified from TG1 cells and transformed in *E. coli* WK6 cells. The nanobodies in pMECS expressed in WK6 cells contain a C-terminal haemagglutinin (HA) and a hexahistidine (His_6_) tag; and were purified from the periplasmic extracts by IMAC and SEC (Figure 1). One single band (except for Nb29) is seen upon Coomassie blue staining of the SDS-PAGE of these nanobodies. The apparent MW of each Nb is close to 15,000–16,000 as expected (Supplementary Materials, Table S1) and the same bands were also revealed after western blot using anti-His antibodies as probe (data not shown).

### 2.3. Specificity of Nanobodies Assessed via Western Blot

Remarkably, the western blot experiments using the nanobodies as a probe confirmed that all selected nanobodies bind to the rStx2aB separated on a gel under non-reducing conditions (data not shown). Treating the rStx2aB with reducing agents (e.g., mercapto-ethanol) before separation on gel, abrogated their recognition by the nanobodies in western blot (data not shown). Conversely, using the rStx2aB protein as a probe in a western blot where the nanobodies were separated (under non-reducing conditions) proved that all five nanobodies interact specifically with rStx2aB (Supplementary Materials, Figure S2).

### 2.4. Interaction between Nbs and rStx2aB Measured via Surface Plasmon Resonance 

#### 2.4.1. In Vivo Biotinylation of rStx2aB Protein

Next, we decided to investigate the Nb–rStx2aB interaction via surface plasmon resonance (SPR). We encountered difficulties trying to immobilize the rStx2aB via chemical coupling onto the sensor chip and decided to capture biotinylated-rStx2aB on a streptavidin-coated chip instead. The rStx2aB protein was therefore cloned with a human IgA1 hinge and a biotin activation domain (BAD) for in vivo biotinylation. The biotinylated-rStx2aB was purified to homogeneity after chromatography on mutein and size exclusion columns (Supplementary Materials, Figure S3). The purified biotinylated-rStx2aB, also eluting as a pentamer after SEC, was captured on the streptavidin-coated sensor chip and served as the ligand to monitor its interaction with the selected anti-rStx2aB Nbs via SPR.

#### 2.4.2. Affinity Measurement

The kinetic on-rates and off-rates of binding between the nanobodies and rStx2aB pentamer were determined from the analysis of the sensorgrams and used to calculate the equilibrium dissociation constant (K_D_) (Table 1). The K_D_ values ranged from 350 nM (Nb29) to 9.6 nM (Nb113) for the monovalent nanobodies. Remarkably the observed R_max_ values for Nb29 and Nb31 reached ~300–350 RU (resonance units), whereas those for Nbs 41, 113 and 140 reached around 800 RU. This suggests that at saturation, up to five molecules of Nb41, Nb113 or Nb140 interact with the rStx2aB pentamer, while only two copies of Nb29 or Nb31 can associate simultaneously on the rStx2aB pentamer.

The binder with highest affinity is definitely Nb113, and so we produced a bivalent Nb113 construct as it is expected to have an improved functional affinity [35]. Two constructs were made. A first construct comprised a tandem repeat of Nb113 separated by a flexible (G_4_S)_3_ linker. The second construct consists of the tandem repeated Nb113 fused with a Nb directed against serum albumin, referred to as trimeric Nb113_2_–NbSA1. The apparent affinity of the bivalent Nb113_2_ increased over 50-fold as compared to the monomeric Nb113, probably due to avidity effects. The functional affinity increased from 9.6 nM to 0.17 nM, which was obtained from an approximately 25-fold higher on rate of binding and from a 2.4-fold slower off rate (Supplementary Materials, Figure S4) (Table 1). The trimeric Nb113_2_–NbSA1 construct displays very similar binding kinetics to that of the bivalent Nb113_2_ construct. The functional affinities for the trimeric and bivalent constructs are 0.25 and 0.17 nM, respectively.

Relative to the R_max_ values of saturating amounts of the monomeric Nb113 (~750 RU), the bivalent Nb113_2_ increased slightly to about 810 RU and the trimeric Nb113_2_–NbSA1 increased significantly to ~1100 RU. This indicates that, per rStx2aB pentamer captured on the chip, on average about four monomeric Nb113 molecules are associated, or two molecules of the Nb113_2_, or two molecules of trimeric Nb113_2_–NbSA1. Indeed, in such instance, the presence of two trimeric molecules instead of two bivalent molecules captured on an rStx2aB pentamer would increase the mass by 30%, as is observed here. 

#### 2.4.3. Epitope Binning

SPR is an elegant technique for epitope binning. By first saturating the Nb binding sites on the biotinylated rStx2aB pentamer with one Nb and then adding a mixture of the same Nb with another Nb it is possible to identify whether those Nbs compete for an overlapping epitope. This exercise performed with all possible Nb pairs confirmed that the binding of Nb29 and Nb31 is mutually exclusive (Supplementary Materials, Figure S5D). Likewise, the association of Nb41, Nb141 and Nb113 are also competing for an overlapping epitope. Interestingly, none of the Nb pairs can saturate their binding sites simultaneously as the observed R_max_ values are never the sum of the independent observed R_max_ values. Surprisingly, if five Nb140 molecules are bound to the rStx2aB pentamer prior to the challenge with a (Nb140 + Nb29) mixture, then all five Nb140 will be outcompeted and replaced by two Nb29 molecules per pentamer (Supplementary Materials, Figure S5E). On the other hand, if two Nb31 molecules are bound per pentamer before adding the (Nb31 + Nb140) mixture, then up to five Nbs can associate with the pentamer (Supplementary Materials, Figure S5F). 

The outcome with Nb113 is more complex under our experimental conditions. For example, once Nb113s are fully occupying their epitopes on the pentamer, then flowing an excess of Nb29 over the chip will displace two Nb113 molecules to associate one Nb29 molecule per rStx2aB pentamer (the observed R_max_ will reach an intermediate value in between those for Nb113 and Nb29; Supplementary Materials, Figure S5A). However, the challenge with the Nb31 fails to replace the prior bound Nb113 molecules on the pentamer (R_max_ and k_off_ traces remain unchanged; Supplementary Materials, Figure S5B).

In conclusion, the epitope binning experiments indicate that two Nb31 molecules (or Nb29) are competing with five Nb113 molecules (or Nb41 or Nb140) for binding to the rStx2aB pentamer. Evidently, since the binding parameters of Nb113 are superior to those of other Nbs, then it is expected that at equal concentrations, Nb113 will eventually manage to replace all other Nbs. For this reason, Nb113 became our lead for crystallization and neutralization studies.

### 2.5. Crystal Structure of Nb113–rStx2aB Complex

**Figure 2:**
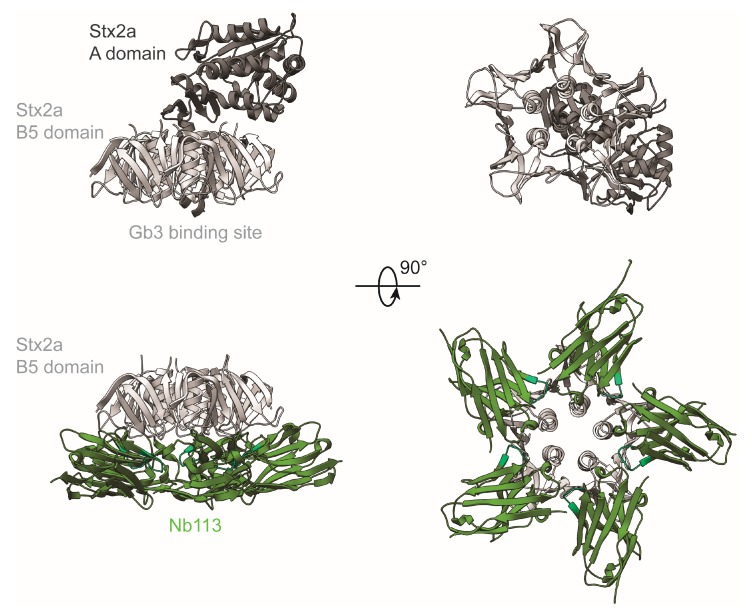
Crystal structure of the Nb113–rStx2aB complex. The top and bottom panels display cartoon representations of the Stx2a holotoxin (PDB ID 2GA4, [14]) and the Nb113–rStx2aB complex (PDB ID 6FE4, this work), respectively. The Stx2a A and B5 domains are colored dim and light grey, respectively. Nb113 is displayed in green.

**Figure 3:**
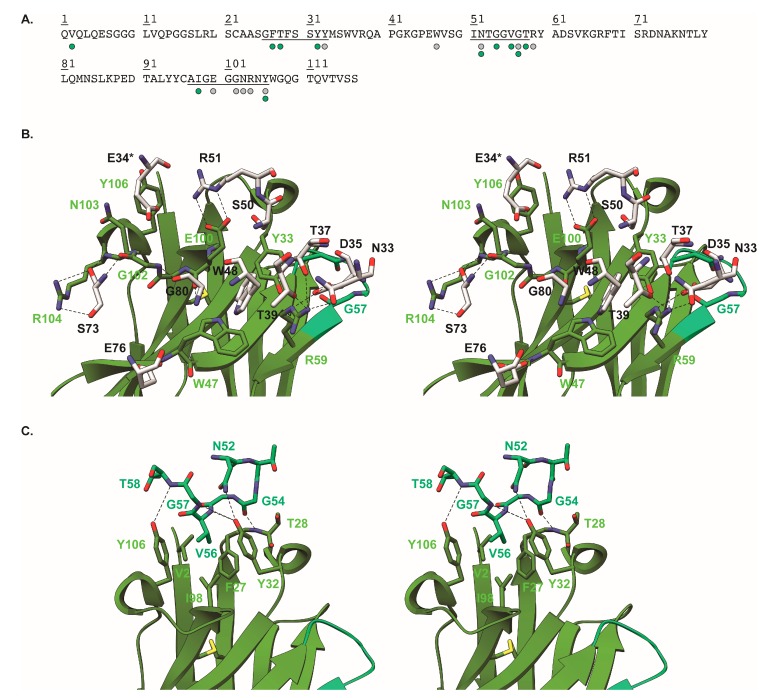
Detailed view of Nb113–rStx2aB and Nb113–Nb113 interactions. (**A**) Amino acid sequence of Nb113 (numbered sequentially). The CDRs are underlined. The residues marked by the green and grey circles are involved in Nb113 and rStx2aB binding, respectively. (**B**) Stereo view of Nb113–rStx2aB interactions. Nb113 is depicted in cartoon representation. For reasons of clarity, only the rStx2aB residues forming part of the epitope of Nb113 are shown and colored in light grey. All interacting residues are shown in a stick representation and are indicated by the colored labels (green and black for Nb113 and rStx2aB residues, respectively). The dashed lines indicate hydrogen bonds or salt bridges (also see Supplementary Materials, Table S3). Residues indicated by an asterisk originate from a neighboring Stx2a B subunit. (**C**) Stereo view of Nb113–Nb113 interactions. The first Nb113 copy is colored as in (**B**). For reasons of clarity only the CDR2 residues of the neighboring Nb113 copy are shown and colored in spring green. All interacting residues are shown in a stick representation and are indicated by the colored labels (green and spring green, respectively). The dashed lines indicate hydrogen bonds or salt bridges (also see Supplementary Materials, Table S4).

To understand the molecular details of the interaction between Nb113 and the rStx2aB pentamer, we determined the structure of the Nb113–rStx2aB complex via X-ray crystallography. The details of the crystallographic experiment are summarized in Supplementary Materials, Table S2. The crystal structure reveals that five copies of Nb113 are bound to the pentameric Stx2a B5 domain (Figure 2). Furthermore, the Nb113 molecules and the Stx2a A subunit are located on the opposite sides of the Stx2 B5 domain (Figure 2). The association of Nb113 to rStx2aB is mediated by Nb113 residues from both, the complementarity determining and framework regions (CDR and FR). More precisely, the Nb113 paratope consists of amino acids from CDR1, FR2, CDR2, FR3, and CDR3 (Figure 3 and Supplementary Materials, Table S3). Noteworthy “interaction hot spots” are rStx2aB Trp48 and Nb113 Arg59. The rStx2aB Trp48 is accommodated by a hydrophobic pocket delineated by Nb113 residues Tyr33, Trp47, Arg59, and Glu100, while Nb113 Arg59 engages in hydrogen bonds and salt bridges with rStx2aB amino acids Asn33, Asp35, Thr37, and Thr39 (Figure 3 and Supplementary Materials, Table S3). Interestingly, most of the residues located in Nb113 CDRs are involved in interactions between two adjacent Nb113 molecules in the Nb113–rStx2aB complex. Almost all CDR2 amino acids are contacted by an amalgamation of FR1, CDR1, and CDR3 residues from a neighboring Nb113 copy (Figure 3 and Supplementary Materials, Table S4). Especially Val56 seems to be the “interaction hot spot” here, as it is the center of a hydrophobic network complemented by Val2, Phe27, Tyr32, Ile98, and Tyr106.

**Figure 4:**
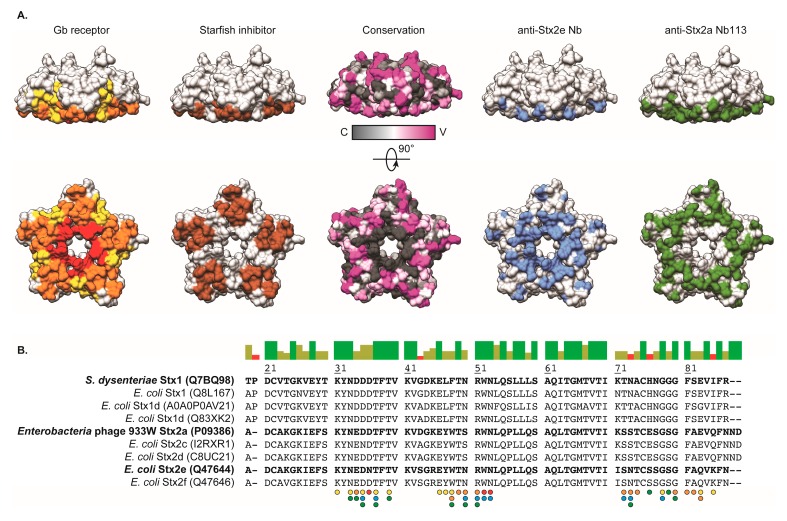
Comparison of Stx ligand and inhibitor binding sites. (**A**) Mapping of ligand and inhibitor binding sites onto a surface representation of the Stx2a B5 domain in the following order from left to right: Gb3 receptor site, STARFISH inhibitor site [23], CONSURF conservation scores, the anti-Stx2e Nb epitope [30] and the anti-Stx2a Nb113 epitope. For Gb3 receptor binding, sites 1, 2, and 3 are colored yellow, orange, and red, respectively. The STARFISH inhibitor pocket corresponds to binding site 2 and is shown in brown. The CONSURF conservation scores are indicated in a gradient from dark grey (conserved, “C”) to magenta (variable, “V”). Finally, the anti-Stx2e Nb and anti-Stx2a Nb113 epitopes are highlighted in blue and green, respectively. The orientations of the Stx2a B5 domain are identical to those in Figure 2. (**B**) Sequence alignment between Stx variants. The sequences in bold are of specific interest and the Uniprot accession codes have been indicated for convenience. The colored bars above the sequence alignment represent the percentage of sequence identity: green (100%), green-brown (between 30% and 100%), and red (below 30%). The residues marked by the colored circles are involved in ligand and/or inhibitor binding. The color codes for the circles are identical to (**A**).

The potential of Nb113 to inhibit Stx2 toxicity on Vero cells is predicted by the observation that the Nb113 epitope overlaps with the toxin’s Gb3 binding site (Figure 4). The Stx2a holotoxin recognizes the Gb3 receptor with the portion of the Stx2a B5 domain located on the opposite side of the anchorage site of the Stx2a A subunit (Figure 2). The surface area dedicated to Gb3 receptor binding consists of three distinct sites, which display a certain degree of sequence variation within the B subunits of the Stx family that explains the Gb3 receptor specificity and thus host cell specificity. The binding pockets of known inhibitors for Stx toxicity overlap with one or more of the Gb3 interaction sites (Figure 4). For instance, the general Stx (Stx1 and Stx2) small-molecule inhibitor STARFISH [23] targets Gb3 binding site 2. The Nb generated against Stx2e (pig edema toxin), associated with clinical cases in pigs [30], blocks Stx2e activity by interacting with residues from all three Gb3 binding sites. For Stx2a neutralization by Nb113, a comparison between the Gb3 interaction sites and the Nb113 epitope on Stx2a reveals that Nb113 acts as a toxin inhibitor mainly by contacting residues located in Gb3 binding sites 1 and 2 (Figure 4). Interestingly, despite their highly overlapping epitopes, the Nb described by Lo and co-workers [30] and Nb113 are not cross-reactive as observed in cytotoxicity assays where Nb113 was unable to neutralize the cytotoxic activity of Stx2e toxin on Vero cells (data not shown). A comparison of the Stx2a and Stx2e sequences shows that, out of all toxin residues contacted by the Nbs, only three differ: Ser50 (Asn for Stx2e), Ser73 (Asn for Stx2e), and Glu76 (Ser for Stx2e). Out of these three, only Ser/Asn50 and Ser/Asn73 are contacted by both Nbs (Figure 4). These mutations might be sufficient to abrogate cross-reactive toxin recognition by Nbs, or at least severely weaken the binding affinity.

### 2.6. Multimerization of Nanobodies and Neutralization of Cytotoxicity in Cell Cultures

**Figure 5:**
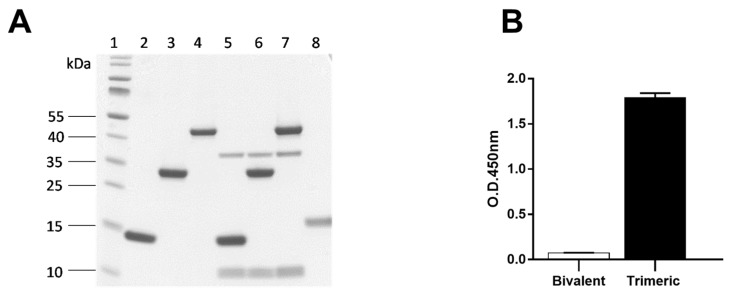
(**A**) Immunocapturing assay. Lane 1: protein MW ladder; Lanes 2–4: Ni^2+^-loaded magnetic beads incubated with His_6_ tagged monovalent Nb113, bivalent Nb113_2_ and trimeric Nb113_2_–NbSA1 alone, respectively; Lanes 5–7: Ni^2+^-loaded magnetic beads incubated with bacterial lysate containing Stx2a toxin plus His_6_ tagged monovalent Nb113, bivalent Nb113_2_ and trimeric Nb113_2_–NbSA1, respectively; Lane 8: Ni^2+^-loaded magnetic beads incubated with the same bacterial extract plus a nanobody against a non *E. coli* antigen (control). (**B**). Serum albumin-binding assay of bivalent Nb113_2_ used as a non-binding control, and albumin-binding trimeric Nb113_2_–NbSA1.

Three different Nb constructs were generated: a monovalent Nb113, a tandem repeat, bivalent Nb113_2_ and a trimeric Nb113_2_–NbSA1 comprising a bivalent Nb113_2_ followed by a Nb against serum albumin. These were expressed with a His_6_ tag and purified to homogeneity by IMAC and SEC (Figure 5A, Lanes 2–4).

#### 2.6.1. Specificity of Bivalent Nb113_2_ and Trimeric Nb113_2_–NbSA1

To prove the specificity of Nb113, bivalent Nb113_2_ and the trimeric Nb113_2_–NbSA1 for Stx2 we performed an immune capture assay on crude periplasmic extract of bacterial cultures from strains expressing Stx2a (Figure 5A) and Stx2c toxins (data not shown). Separating the captured molecules by SDS-PAGE and staining by Coomassie clearly indicated that Stx comprising Stx2a A (band at 36 kDa) and Stx2a B subunits (band at 10 kDa) were associated to the Nb constructs (Figure 5A, lanes 5, 6, 7). No other bacterial proteins were observed. The use of a Nb directed against a mammalian protein target fails to capture any bacterial protein (Figure 5A, lane 8).

To demonstrate that the Nb against serum albumin (NbSA1) was able to recognize mouse serum albumin, we performed an ELISA using mouse serum albumin coated in wells of microtiter plates. Whereas the bivalent Nb113_2_ fails to recognize the serum albumin in ELISA, the trimeric Nb113_2_–NbSA1 definitely recognizes the serum albumin (Figure 5B).

#### 2.6.2. Neutralization of Stx2

**Figure 6:**
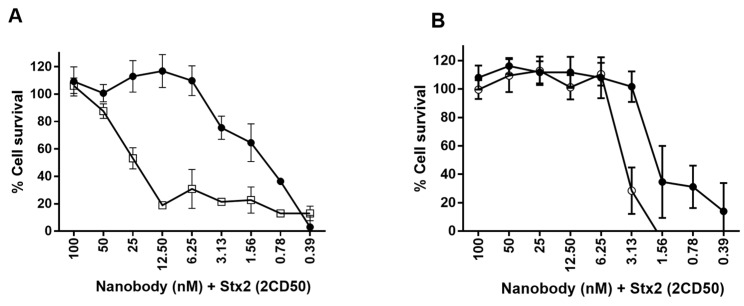
Neutralization of Stx2a cytotoxicity by nanobody constructs on Vero cells. (**A**) The monovalent Nb113 (open squares) and bivalent Nb113_2_ (filled circles) were tested at different doses to neutralize 2 CD_50_ of Stx2a. (**B**) The bivalent Nb113_2_ (filled circles) and trimeric Nb113_2_–NbSA1 (open circles) were tested at different doses to neutralize 2 CD_50_ of Stx2a.

Finally, the neutralization of Stx2 (derived from bacterial periplasmic extracts) by the various Nb constructs was tested in a Vero cell assay. In an initial experiment Nb29, Nb31, Nb41, Nb113 and Nb140 were evaluated separately in their capacity to protect Vero cells from Stx2 damage. Only Nb113 protects Vero cells against the Stx2a and Stx2c toxicity in a concentration dependent manner, but not against the Stx2e toxin (data not shown). Since the multivalent Nb113 constructs display a higher affinity towards rStx2aB compared to monovalent Nb113 (*cfr.* SPR), we compared the neutralization capacity of all Nb113 constructs (Figure 6A). It is evident that the bivalent construct showed a significant increased neutralizing capacity. In a second comparison, we evaluated the neutralization capacity of the bivalent Nb113_2_ and the trimeric Nb113_2_–NbSA1 (Figure 6B). Clearly, the trimeric format neutralizes the Stx2a, although slightly less well than the bivalent Nb113_2_. These analyses demonstrate that a larger number of cells survive at lower concentration of the bivalent Nb113_2_ in line with its higher avidity and that the fusion of the NbSA1 to the dimeric nanobody does not significantly affect the neutralizing capacity.

## 3. Discussion

The large outbreak of STEC-HUS in Germany in 2011 [36] exposed the lack of effective treatments. A solution to this health problem could be offered using recombinant single domain antibodies, referred to as nanobodies, which currently constitute a highly versatile and efficient alternative for the development of therapeutic agents [37,38]. Nanobodies are of interest due to their small size favoring rapid bioavailability and fast delivery to sites of toxin uptake [39,40,41]. In the last couple of years, several groups have generated nanobodies against Stx2 toxins. Tremblay et al. [28] described the generation of VHH libraries from an alpaca immunized with formalin-inactivated Stx1 and Stx2 toxoids, while Mejias et al. [29,42] used an immunogen consisting of the B subunit of Stx2 fused at its amino terminal to the lumazine synthase from *Brucella* spp. (BLS-Stx2B). Both groups have demonstrated that Stx-inhibiting Nbs can be employed to prevent Stx-induced disease and lethality in experimental mouse models [28,29]. In the present work, we immunized an alpaca with the recombinant B moiety of Stx2a (rStx2aB), which is considerably easier to produce than toxoids. We then constructed a library of nanobodies from this animal and retrieved target specific nanobodies after phage display and three rounds of panning on immobilized rStx2aB. In silico analysis of the amino acid sequences of these binders revealed the presence of four families of nanobodies. At least one representative nanobody (named Nb29, Nb31, Nb41, Nb113 and Nb140) of each family was expressed (Nb41 and Nb140 belong to the same family), purified to homogeneity and shown to bind specifically to the rStx2aB protein, as demonstrated by ELISA, Western blot and immunocapturing. 

The selected Nbs bind the rStx2aB antigen with nanomolar affinity and seem to target overlapping epitopes as evidenced by SPR. Remarkably, out of five available epitopes on the rStx2aB pentamer, only two could be occupied by Nb29 and Nb31, while the other Nbs (Nb41, Nb140 and Nb113) could completely saturate rStx2aB (i.e., up to five Nbs per rStx2aB pentamer). 

From these Nbs, Nb113 has been identified as our lead compound through a combination of techniques. SPR revealed that, with an affinity of 9.6 nM, Nb113 was by far the best rStx2aB binder in our collection, while the ability of Nb113 to recognize and interact with native Stx2a holotoxin was confirmed by immunocapturing assays. Finally, in vitro toxicity assays have demonstrated that Nb113 can neutralize Stx2a-induced death of Vero cells. Determining the structure of the Nb113–rStx2aB complex by X-ray crystallography unraveled the molecular basis for Nb113-mediated neutralization of Stx2a toxicity. The Nb113–rStx2aB crystal structure shows that five Nb113 molecules are bound to the Stx2a B pentamer, corroborating the SPR data. The epitope targeted by Nb113 largely overlaps with a portion of the Gb3 receptor site (sites 1 and 2). The occupation of all five Gb3 binding sites would indeed be expected to impede the interaction between the Stx2a B5 pentamer and its target receptor and, hence, alleviate Stx2a toxin cell entry and toxicity.

Based on the lead compound Nb113, two multivalent variants were generated: bivalent Nb113_2_ and trivalent Nb113_2_–NbSA1. Indeed, the strict monomeric behavior of nanobodies facilitates the production of oligomerized formats. Nanobodies can be dimerized either with identical or different VHH fragments to obtain bivalent, biparatopic or bispecific constructs that can lead to a broader neutralization capacity due to avidity or chelating effects [37]. Although the expression of oligomeric nanobodies is slightly less efficient compared to the monomeric version, yields are generally more satisfactory than those for dimeric constructs made with single chain Fvs form conventional antibodies where domain mispairing can occur [33,38,43]. The SPR measurements confirm that Nb113_2_ and Nb113_2_–NbSA1 bind to the rStx2aB pentamer with approximately 50-fold higher affinity compared to the monomeric nanobody format. The higher affinity was obtained by the combined effect of an approximately 25-fold higher kinetic on rate and a 2.5-fold slower off rate. Moreover, both formats were also shown to capture the whole Stx2 holotoxin from crude periplasmic extracts from *E. coli* strains expressing native Stx2a and Stx2c variants at least as efficiently as monovalent Nb113. In accordance with the SPR experiments, we demonstrated that oligomerization of the Nb113 exerted a positive effect on the neutralizing capacity. For example, the neutralization dose could be lowered from 100 nM for the monovalent Nb113 to 6.25 nM for the bivalent Nb113_2_. In the same way, the trimeric bi-specific Nb113_2_–NbSA1 exhibited a similar neutralization profile compared to bivalent Nb113_2_, confirming the enhanced neutralization capacity of the Stx2 toxin activity by dimerizing Nb113. Similar neutralization capacities were also noticed for the multimerized Nb constructs generated in previous studies [28,29,30]. Although Nb113 was able to fully neutralize the Stx2-induced toxicity, due to its specificity for Stx2a and Stx2c, it failed to neutralize Stx2e, which causes edema disease in pigs (data not shown). The improved neutralization potential of bivalent Nb113_2_ constructs is also supported by the Nb113–rStx2aB crystal structure. The (G_4_S)_3_ linker employed in the Nb113_2_ and Nb113_2_–NbSA1 constructs is sufficiently long and flexible to allow the association of both Nb113 entities with two neighboring B domains of a Stx2a B pentamer.

In view of the above-mentioned findings, we think that the Nb113_2_ and Nb113_2_–NbSA1 constructs will be good leads for future therapeutic investigations. However, compared to the trimeric construct, it is predicted that larger quantities of Nb113_2_ will be needed to treat an infection. The small size of bivalent Nbs makes that they are cleared rapidly from blood via the kidneys. Hence, any increase in the blood retention will improve the performance of the therapeutic compound. Increased blood retention should be obtained by fusing Nb113_2_ with NbSA1 (a serum albumin specific Nb, which is cross-reactive between human and mouse serum albumin but does not bind to bovine serum albumin (Master thesis of Kamil Grzyb at Vrije Universititeit Brussel, 2013)). This strategy has been shown previously to increase the blood retention time [44], and we have shown here that the presence of the NbSA1 at the C-end of the bivalent Nb113_2_ maintains the same affinity and neutralization capacity towards Stx2a as the bivalent Nb113_2_. As demonstrated in the work of Meijas et al., the generation of a trimeric construct containing two copies of a Stx-neutralizing Nb and a Nb targeting serum albumin has the potential to drastically improve treatment outcome [29]. It is expected that the Nb113_2_–NbSA1 construct will allow the initiation of pre-clinical evaluations and, if positive, could eventually be translated to applications for human therapy.

## 4. Materials and Methods 

### 4.1. Ethical Statement

Ethical clearance for alpaca handling was received from VUB ethical committee for animal welfare. The alpaca was under surveillance of veterinarians and subjected to clinical inspection for general good health condition before each immunization and absence of infections.

### 4.2. Immunogen Production

The B subunit of Stx2a (rStx2aB) was produced recombinantly in *E. coli* BL21. Briefly, the gene fragment encoding the B subunit of Stx2 was amplified by PCR using genomic DNA of *E. coli* O157:H7 strain EDL933 (NCBI Reference Sequence: NC_002655.2) as template. The amplified fragment was inserted into the pET22b vector, in frame with a C-terminal 6xHis tag. The insert was confirmed by nucleotide sequencing. The rStx2aB–pET22b construct was transformed into *E. coli* BL21 cells and transformed cells were selected on LB agar plates supplemented with 2% glucose and 100 μg/mL ampicillin. Single colonies from the plates were pre-cultured overnight at 37 °C in LB media supplemented with 100 μg/mL ampicillin. One mL of each pre-culture was used to inoculate a flask containing 300 mL of TB media supplemented with 2% glucose and 100 μ/mL ampicillin. Cells were grown at 37 °C with aeration until they reached the exponential growth phase (O.D._600nm_ 0.6). Expression of recombinant protein was induced by adding isopropyl β-d-1-thiogalactopyranoside (IPTG) up to 1 mM and incubating the cultures overnight at 28 °C with aeration. Cells were harvested by centrifugation. Bacterial pellets were resuspended and the periplasmic proteins were extracted via osmotic shock. The periplasmic extract was loaded on IMAC columns, washed with PBS and the rStx2aB was eluted with 0.5 M imidazole in PBS. The fractions containing the target protein were pooled and concentrated to a final volume of 2 mL for the subsequent SEC step on a Superdex-75 16/60 column (GE Healthcare, Chicago, IL, USA), which was pre-equilibrated with at least one column volume of PBS. The sample was eluted at a flow rate of 1 mL/min. Fractions containing the target protein were pooled and stored at 4 °C. The identity of the purified rStx2aB was confirmed by Western blot (WB) with anti-Stx2 monoclonal antibodies. Aliquots of the purified rStx2aB protein were stored in PBS at −80 °C until further use.

### 4.3. Alpaca Immunization

An alpaca was immunized subcutaneously at weekly intervals during six weeks. The immunogen consisted of rStx2aB protein (100 µg in PBS) mixed with an equal volume of GERBU LQ adjuvant. Four days after the last immunization, blood was collected in anti-coagulating vacuum tubes and transported to the laboratory.

### 4.4. Construction of the Immune Phage Display Library

First, peripheral blood lymphocytes (PBLs) were purified on a density cushion. The total RNA was extracted from the PBLs and cDNA was synthesized by reverse transcription polymerase chain reaction (RT-PCR) using ThermoScript RT-PCR Kit. Then, a multi-step PCR was used to amplify VHH gene fragments. The first PCR step was performed with primers CALL001 (5′-GTCCTGGCTGCTCTTCTACAAGG-3′) and CALL002 (5′-GGTACGTGCTGTTGAACTGTTCC-3′) [45]. The amplicons of ~600 bp were extracted from 1% agarose gel and used as template for the second PCR with A6E (5′-GATGTGCAGCTGCAGGAGTCTGGRGGAGG-3′) (R is G and A) and PMCF: (5′-CTAGTGCGGCCGCTGAGGAGACGGTGACCTGGGT-3′) primers (PstI and NotI restriction enzyme sites are underlined).

The PCR amplicons were purified with GenElute PCR Clean-Up kit (Qiagen) and ligated into the phagemid pMECS (cut with PstI and NotI restriction enzymes) at 16 °C for 16 h with T4 DNA ligase. Electrocompetent suppressor *E. coli* TG1 cells were used as host of the VHH library inserted into the pMECS phage display vector. The VHH repertoire was expressed on filamentous phage after infection of TG1-transformed cells with M13K07 helper phages.

### 4.5. Selection of rStx2aB-Binding Nanobodies

Selection of Nbs against rStx2aB was performed with 100 µL of rStx2aB (100 µg/mL) immobilized in a well of a Maxisorp microplate (Nunc, Thermo Fisher Scientific, Waltham, MA, USA). After the second and third round of panning, single colonies were picked, cultured and induced with 1 mM IPTG to express the cloned Nb in the periplasm. Subsequently, the recombinant Nbs extracted from the periplasm (PE) were tested in an ELISA (PE-ELISA), to identify those clones that produce a Nb recognizing the rStx2aB antigen. The clones giving a positive signal in PE-ELISA were sent for sequencing and their predicted amino acid sequence was analyzed in silico and classified in families according to the amino acid diversity within the CDRs.

### 4.6. Expression and Purification of rStx2aB-Binding Nanobodies

After choosing at least one representative clone from each Nb family, the phagemid was extracted from TG1 cells and transformed into electrocompetent non-suppressor *E. coli* WK6 cells. The transformed cells were selected (overnight at 37 °C) on LB agar plates with 2% glucose and 100 µg/mL ampicillin. A single colony from the plates was cultured in TB medium supplemented with 0.1% (*w/v*) glucose, 100 μg/mL ampicillin and 2 mM MgCl_2_. The Nb expression was induced with 1 mM IPTG when the culture reached an O.D._600nm_ of 0.6, and incubation was continued overnight at 28 °C while shaking. The periplasmic proteins were extracted by osmotic shock and purification of His-tagged Nbs was performed as described before [45].

Purity of Nbs was assessed by SDS-PAGE staining with Coomassie blue. A Western blot using anti-His monoclonal antibodies was used to confirm the identity of the Nb. The concentration of purified Nbs was measured via UV spectrophotometry on NanoDrop 2000 (Thermo Scientific, Waltham, MA, USA), using the extinction coefficient predicted with the ExPASy ProtParam tool [46].

### 4.7. Western Blot Using Nanobodies as Probe

For this assay, rStx2aB protein was run (5 µg per lane) on 12% Bis-Tris SDS-PAGE under reducing and non-reducing conditions. The proteins were transferred onto a nitrocellulose membrane (GE Healthcare) and residual protein binding sites on the membrane were blocked by incubation in 2% skimmed milk in PBS for 1 h at room temperature (RT). Then, the membrane was soaked for 1 h at RT in purified Nb (25 µg/mL) to allow Nb–rStx2aB binding. The membrane was washed three times with PBS, and mouse anti-HA tag monoclonal antibody (1:2000 in blocking buffer) was added and incubated for 1 h at RT to reveal the presence of rStx2aB-bound Nb. Subsequently, the membrane was washed three times with PBS, and goat anti-mouse IgG HRP-conjugated was added (1:2000 in blocking buffer) and incubated for 1 h at RT. Finally, the membrane was washed three times with PBS before adding 18 mg of 4-chloro 1-naphtol in 6 mL ethanol, 30 mL TPA buffer and 18 µL H_2_O_2_ for colorimetric visualization of the rStx2aB bands.

### 4.8. Western Blot Using rStx2aB as Probe

For this assay, each nanobody (5 µg) was run on a 12% Bis-Tris SDS-PAGE (Bio-Rad, Hercules, CA, USA) under non-reducing conditions and transferred to nitrocellulose, after incubation with 2% skimmed milk in PBS, the purified rStx2aB protein was added at 10 µg/mL and incubated for 1 h at RT. The membrane was washed three times with PBS and mouse anti-Stx2 toxin monoclonal antibody (1:2000 in blocking buffer) was added and incubated for 1 h at RT. Subsequently, the membrane was washed three times with PBS before adding goat anti-mouse IgG HRP-conjugated (1:2000 in blocking buffer) and incubated for 1 h at RT. Finally, the membrane was washed three times with PBS before adding 4-chloro 1-naphtol substrate (18 mg) in 6 mL ethanol, 30 mL TPA buffer and 18 µL H_2_O_2_ for colorimetric visualization of the Nb bands.

### 4.9. In Vivo Biotinylation of rStx2aB for Surface Plasmon Resonance Assays

The gene fragment encoding rStx2aB was amplified by PCR and recloned in an expression vector (Amp^R^) to incorporate the human IgA1 hinge and the biotin acceptor domain (BAD) at the C-terminus of the expressed rStx2aB protein [47]. The ligated vector was co-transformed with BirA plasmid (Chloramphenicol^R^), encoding biotin protein ligase, in electrocompetent *E. coli* WK6 cells [47]. To produce biotinylated rStx2aB, 1 mL of an overnight starter culture was inoculated in 330 mL terrific broth (TB) supplemented with ampicillin (100 μg/mL) and chloramphenicol (35 μg/mL). Cells were grown at 37 °C, while shaking, until an O.D._600nm_ of 0.6 was reached, then 0.02 mM D-biotin was added to the cultures and 30 min later, the expression of the recombinant rStx2aB-BAD protein was induced with 0.2 mM IPTG and overnight incubation at 28 °C. The next morning, cells were harvested by centrifugation. The biotinylated rStx2aB from the bacterial lysate was purified by affinity chromatography on streptavidin mutein matrix (Roche, Basel, Switzerland), followed by size exclusion chromatography on FPLC system (ÄKTA, GE Healthcare) [48]. Protein production was analyzed by SDS-PAGE under reducing conditions using 12% Bis-Tris gel (Bio-Rad) and Western blot.

### 4.10. Affinity Measurement and Epitope Binning

To determine the binding properties of Nbs to rStx2aB, an SPR experiment was performed on Biacore T200 (GE Healthcare) according to the manufacturer’s instructions. The CAP sensor chip (GE Healthcare), coated with streptavidin, was used for capturing bioinylated-rStx2aB at a concentration of 10 μg/mL and a flow rate of 10 μL/min for 1 min. This resulted in coupling ~680 RU biotinylated rStx2aB to the sensor chip surface. Meanwhile, one flow cell of the sensor chip was left without captured biotinylated-rStx2aB to provide a reference surface. Nanobodies were prepared at different concentrations starting from 500 nM using a 2-fold serial dilution until 1.95 nM in running buffer (20 mM HEPES, 150 mM NaCl, 0.005% Tween-20, 3.4 mM EDTA, pH 7.4). 

For all Nb constructs tested, sensorgrams for different analyte concentrations (starting from 500 nM using a 2-fold serial dilution) plus a 0 concentration (injection of running buffer) were collected at a flow rate of 30 µL/min and a data collection rate of 1 Hz. Analyte injections were performed with association and dissociation phases of 180 s and 300 s, respectively. Prior to data analysis, reference and zero concentration data were subtracted from the sensorgrams. The collected data were fitted according to a 1:1 Langmuir binding model (one Nb to one monomer of rStx2aB) from the three experimental flow cells of a single biosensor chip. All experimental data were treated with Biacore T200 Evaluation Software to calculate the kinetic k-on and k-off rates and the equilibrium dissociation constant (K_D_).

For epitope binning, we first injected an excess of nanobody A (100 times its K_D_ value) for 300 s to saturate all epitopes on the pentameric biotinylated rStx2aB. This was followed by applying a mixture of excess nanobody A and nanobody B (both at a concentration of 100 times their K_D_ value) and monitoring the RU over a period of 300 s. The chip was then injected with buffer without any nanobody for 500 s. All possible Nb combinations were tested including all orders of injection. 

### 4.11. Crystallization, Data Collection, Data Processing, and Structure Determination of Nb113–rStx2aB Complex

The Nb113–rStx2aB complex was generated by mixing Nb113 and rStx2aB at a ratio of 1:1.2 allowing the sample to equilibrate for at least 45 min prior to purification on a Superdex 200 16/60 column (GE Healthcare) pre-equilibrated with at least one column volume of PBS. The sample was eluted at a flow rate of 1 mL/min. Fractions containing the Nb113–rStx2aB complex were pooled and stored at 4 °C.

The Nb113–rStx2aB complex was concentrated to 12.8 mg/mL using a 5000 MWCO concentrator (Vivaspin20, Sartorius, Göttingen, Germany). Crystallization conditions were screened manually using the hanging-drop vapor-diffusion method in 48-well plates (Hampton VDX greased, Aliso Viejo, CA, USA) with drops consisting of 2 μL protein solution and 2 μL reservoir solution equilibrated against 150 μL reservoir solution. Commercial screens from Jena Bioscience (JBScreen Classic 1–4, Jena, Germany) were used for initial screening. The purification tags of both proteins were retained during crystallization. The crystal plates were incubated at 20 °C. Diffraction-quality crystals of the complex were obtained in JBScreen Classic 1 Solution B1 (28% *w*/*v* PEG 400, 100 mM HEPES pH 7.5, 200 mM CaCl_2_) and JBScreen Classic 1 solution B2 (15% *w*/*v* PEG 4000, 100 mM sodium citrate pH 5.6, 200 mM ammonium sulphate), and crystals grew at RT within a couple of hours.

The Nb113–rStx2aB crystals were cryo-cooled in liquid nitrogen with the addition of 25% (*v*/*v*) glycerol to the mother liquor as a cryo-protectant in 5% increments. Data were collected on the i24 beamline at the DIAMOND synchrotron (Didcot, UK). Because the crystals were quite sensitive to beam exposure and lost their diffractive power relatively fast, three data sets were collected from different regions of the same crystal. Five hundred frames were collected for each data set with an oscillation range of 0.1° and starting angles of 0°, 50°, and 100° respectively. The three data sets were processed and merged with XDS [49]. The quality of the collected data set was verified by close inspection of the XDS output files and through phenix.xtriage in the PHENIX package [50]. Twinning tests were also performed by phenix.xtriage. Analysis of the unit-cell contents was performed with the program MATTHEWS_COEF, which is part of the CCP4 package [51]. The structure of the Nb113–rStx2aB complex was determined by molecular replacement with PHASER-MR [52]. Two components were used as search models: (i) the Stx2a B subunit pentamer, which was obtained from the published structure of Stx2a holotoxin (PDB ID: 2GA4, [14]) and (ii) the structure of the anti-furin Nb (PDB ID: 5JMR, [53]) of which the CDRs had been deleted for the molecular replacement. A search for 1 and 5 copies of the first and second component, respectively, provided a single solution (top TFZ = 25.9 and top LLG = 2506.815). From here, refinement cycles using the maximum likelihood target function cycles of *phenix.refine* [54] were alternated with manual building using Coot [55]. During the refinement process, the PDB_REDO server [56] and BUSTER [57] was also employed. The final refinement cycle included TLS (Translation/Libration/Screw) refinement, for which the optimal TLS groups were determined using the TLSMD Web server [58]. The crystallographic data for the Nb113–rStx2aB complex are summarized in Supplementary Materials, Table S2 and have been deposited in the PDB (ID 6FE4). Molecular graphics and analyses were performed with UCSF Chimera [59]. CONSURF scores were calculated using the CONSURF server [60] and were based on a multiple sequence alignment of 53 Stx variants.

### 4.12. Construction of a Bivalent Nanobody

First, the plasmid encoding the Nb113 was digested with PstI and BstEII restriction enzymes and the gene fragment containing the Nb113 sequence was recloned into the pHEN6c vector for monovalent Nb expression with His6 tag and without the HA-tag (Nb113 monovalent). Next a PCR was performed using the cloned monovalent Nb113 (Nb113 pHEN6c) as template to amplify the Nb113 gene and adding a downstream (Gly_4_Ser)_3_ encoding linker. The primer annealing at the 5′ end of the Nb113 gene also removed the PstI site and enforces a restriction enzyme site for NcoI. Afterwards, both the generated amplicon as well as the Nb113 pHEN6c were digested using NcoI and PstI, and the digested products cleaned and finally ligated using T4 DNA Ligase (Thermo Fisher Scientific).

The ligation product was transformed in *E. coli* WK6 electrocompetent cells, plated on LB agar containing glucose (2%) and ampicillin (100 µg/mL), and incubated at 37 °C overnight. A few colonies were picked individually and PCR was performed to ensure the presence of an insert with a size that corresponds to the tandem Nb113 genes separated by the (Gly_4_Ser)_3_ linker. The PCR-positive colonies were cultured in LB medium and their plasmids extracted and sent for sequencing to confirm the presence of the correct insert.

### 4.13. Construction of Trimeric Bispecific Nb113_2_–NbSA1

To increase the serum half-life of the bivalent Nb113_2_, a nanobody that binds to serum albumin of mice and humans (NbSA1) was fused at the C-terminal end of the bivalent Nb113_2_. This construction was built using the “splicing by overlap” PCR technique to fuse the NbSA1 preceded by a (Gly_4_Ser)_3_ linker.

### 4.14. Expression and Purification of Multimerised Nanobodies

The expression of bivalent Nb113_2_ and trimeric Nb113_2_–NbSA1 was exactly as explained for the monomeric Nb113 multimerized nanobodies (monovalent Nb113, bivalent and trimeric-bispecific) were purified according to the same protocols described previously (see Section 4.6).

### 4.15. Immunocapturing Experiments

For this assay, 25 µg of monovalent, bivalent and trimeric nanobodies (in PBS) were mixed with 200 µL of periplasmic extract of bacterial cultures from strains expressing Stx2a and Stx2c toxins. These mixtures were incubated for 30 min at RT and subsequently used for the immunocapturing assay using the QuickPick™ IMAC Kit (Bionobile, Pargas, Finland), according to the manufacturer’s instructions. This kit contains magnetic particles suitable for binding and purification of His-tagged proteins from cell extracts [61]. The eluate was loaded on a 12% bis-tris acrylamide precast gel (Bio-Rad) and stained with either Coomassie Blue or silver. The nanobodies used in this assay are His-tagged, but the Stx2 toxins are not. As controls we used the nanobodies incubated with the magnetic particles only and as negative control a nanobody, targeting a different irrelevant protein, incubated with the Stx2a extract.

### 4.16. Serum Albumin Binding ELISA

This assay was done to assess the binding of trimeric Nb113_2_–NbSA1 to mouse serum albumin. Several wells of a ninety-six well microtiter plate were coated with mouse serum (1:100 in Na_2_CO_3_ pH 8.6) at 4 °C overnight. Residual protein binding sites on plates were blocked with skimmed milk (2% in PBS) and incubated at RT for 1 h. The plates were washed three times with PBS and incubated with 100 μL of nanobodies (10 µg/mL of either bivalent Nb113_2_ or trimeric Nb113_2_–NbSA1 for 1 h at RT. The plates were washed three times with PBS-T and incubated with 100 μL of biotinylated anti-His antibody (1:2000 in PBS) for 1 h at RT. Then, the plates were washed three times before adding 100 μL streptavidin-HRP conjugate and incubating at RT for 1 h. Finally, the plates were washed six times with PBS-Tween and the colorimetric substrate 3,3′,5,5′-tetramethylbenzidine (TMB) was added and plates were kept in the dark until color developed. The reaction was stopped by the addition of 100 μL of 3 M HCl, and absorbance was measured at 450 nm with a microplate reader (Molecular Devices, San Jose, CA, USA).

### 4.17. Neutralization of Stx2 Cytotoxicity in Cell Cultures

The Stx2 toxin-neutralizing activity of the selected nanobodies was assessed in a cell-based assay using Vero cells (African green monkey kidney cells), as described previously [62]. Briefly, two-fold serial dilutions of Nbs in PBS, with a starting concentration of 100 nM, were incubated at RT for 30 min in presence of an equal volume of 2 CD_50_ Stx2 toxin per well. The Stx2 toxin/nanobody mixtures were added to wells of a 96-well plate (Falcon BD) containing a confluent culture of Vero cells (ATCC CCL-81) grown in Dulbecco’s modified Eagle’s medium (DMEM), supplemented with 5% fetal bovine serum. After 24 h incubation in a humidified incubator (5% CO_2_ environment), the medium was removed, and the remaining cells were fixed with a solution of 2% formaldehyde in PBS for 10 min. Cells were then stained with 0.13% (*w/v*) crystal violet dissolved in 5% ethanol, 0.2% formaldehyde in PBS for 10 min. Cells were washed six times with water. Cell-adsorbed crystal violet was extracted in 200 µL of 50% ethanol in PBS, and absorbance was measured at 595 nm. Nanobody-only and toxin-only controls were included in the assays. The results were expressed as percentage viability compared with that of control Vero cells in presence of nanobody without toxin (100% viability) and with toxin only (0% viability) [30].

## Figures and Tables

**Table 1:** Kinetic parameters of Nb–rStx2aB interactions obtained via fitting the collected SPR data.

	k_on_ (10^5^ M^−1^s^−1^)	k_off_ (s^−1^)	K_D_ (nM)	*Χ*^2^	R_max_ Expected (RU)	R_max_ Observed (RU)	*n* ^3^
Nb29	8.7	0.2998	344.4	3.33	173	357	2.06
Nb31	11.38	0.0812	71.33	3.58	163	310	1.90
Nb41	1.26	0.0049	39.01	51.5	182	805	4.42
Nb140	1.32	0.0071	53.65	38.6	181	852	4.70
Nb113	1.95	0.0019	9.6	27.0	166	748	4.52
Nb113_2_^1^	48.27	0.0008	0.17	18.4	302	813	2.69
Nb113_2_-SA1 ^2^	34.15	0.0009	0.25	50.6	466	1114	2.39

^1^ Bivalent Nb construct. ^2^ Trimeric construct of bivalent Nb113_2_ fused to Nb-SA1. ^3^ *n* = (R_max,observed_)/(R_L_ (MM_Nb_/MM_pentameric rStx2aB_)) = (R_max,observed_)/(R_max,expected_), where R_L_ represents the amount of biotinylated rStx2aB immobilized on the sensor chip surface (680.1 RU).

## Supplementary Materials

The following are available online at http://www.mdpi.com/2072-6651/10/3/108/s1, Figure S1: Quality of rStx2aB preparation, Figure S2: Western blot band pattern of purified nanobodies developed with rStx2aB protein as a probe, Figure S3: Biotinylated rStx2aB, Figure S4: SPR sensorgrams of monovalent Nb113, bivalent Nb113_2_ and trimeric Nb113_2_–NbSA1 on biotinylated rStx2aB, Figure S5: Epitope binning, Table S1: Main biochemical properties of rStx2aB-specific nanobodies and derivatives as calculated from Expasy ProtParam, Table S2: Data collection and refinement statistics. Statistics for the highest resolution shall are shown in parentheses, Table S3: List of interactions between Nb113 and rStx2aB, Table S4: List of interactions between neighboring Nb113 molecules in the Nb113–rStx2aB complex.
