# Supplementary material for: Structural Basis for the Specific Neutralization of Stx2a with a Camelid Single Domain Antibody Fragment
Source: Toxins (Basel). 2018 Mar 1;10(3):108. doi: 10.3390/toxins10030108 (PMC5869396; doi:10.3390/toxins10030108)
Supplement: Supplementary file 1 [file toxins-10-00108-s001.pdf]

Supplementary Materials: Structural Basis for the Specific Neutralization of Stx2a with a Camelid Single Domain Antibody Fragment

Robert Alvin Bernedo-Navarro, Ema Romão, Tomomasa Yano, Joar Pinto, Henri De Greve, Yann G.-J. Sterckx and Serge Muyldermans

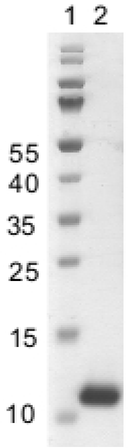

Figure S1. Quality of rStx2aB preparation. SDS-PAGE of purified rStx2aB stained with Coomassie blue. This preparation was used as immunogen in alpaca. Lane 1: Protein size marker with molecular mass in kDa (left); Lane 2: purified rStx2aB protein, respectively.

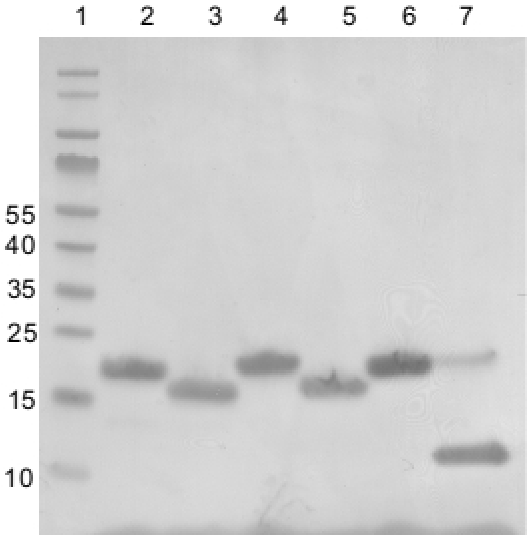

Figure S2. Western blot band pattern of purified nanobodies developed with rStx2aB protein as a probe. Lane1: protein size marker with molecular mass in kDa (left); Lanes 2 to 6: Nb29, Nb31, Nb41, Nb113 and Nb140, Lane 7: rStx2aB respectively.

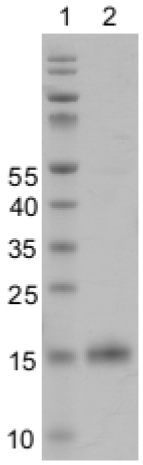

Figure S3. Biotinylated rStx2aB. SDS-PAGE of purified biotinylated-rStx2B used to bind on streptavidin coated sensor chips. Lane 1: Protein size marker with molecular mass in kDa (left); Lane 2: purified biotin-rStx2aB protein, respectively.

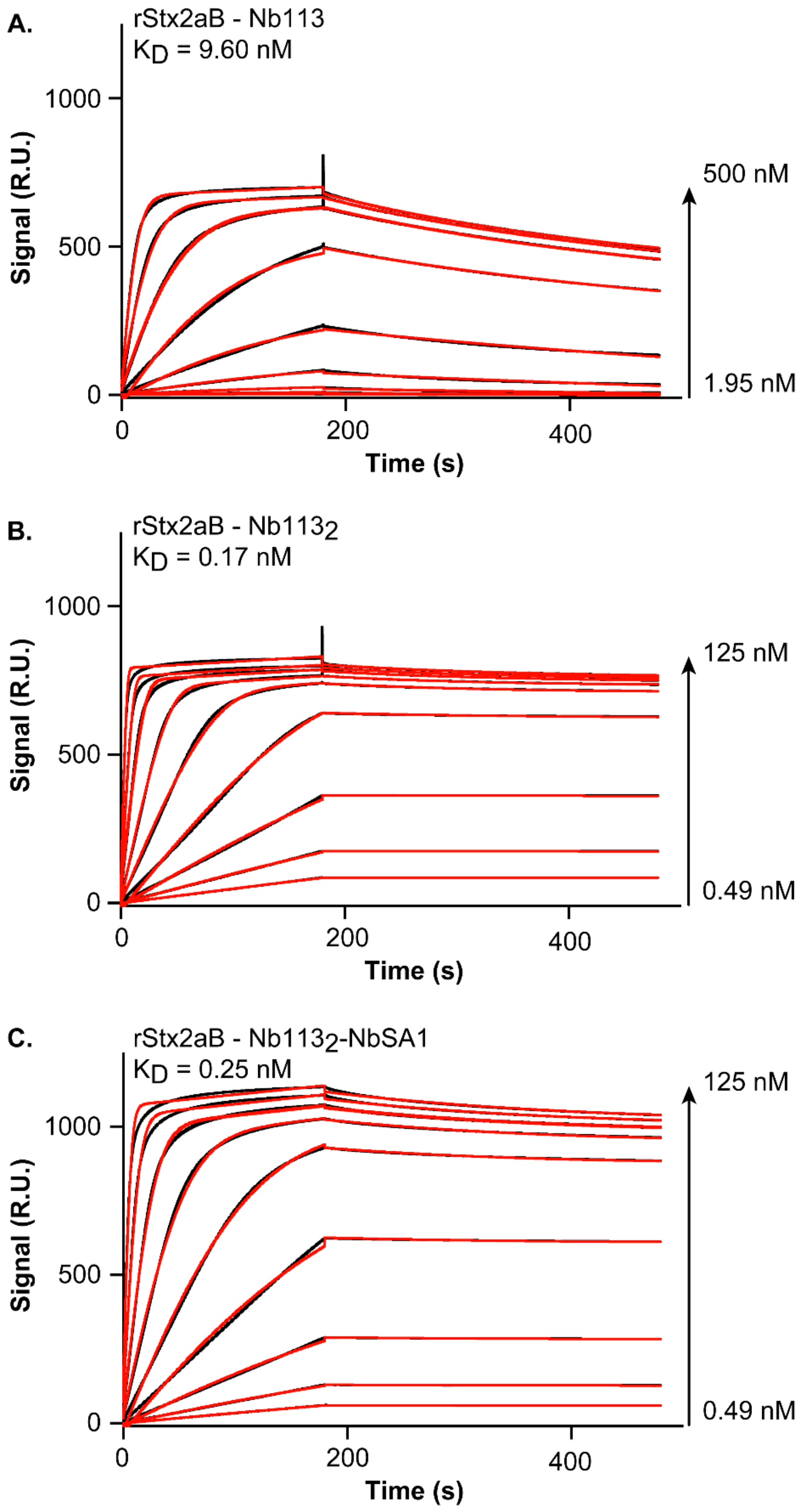

Figure S4. SPR sensorgrams of monovalent Nb113, bivalent $Nb113_2$ and trimeric $Nb113_2$-SA1 on biotinylated rStx2aB. (**A**) Sensorgram of Nb113; (**B**) sensorgram of bivalent $Nb113_2$ and (**C**) sensorgram of trimeric $Nb113_2$-SA1.

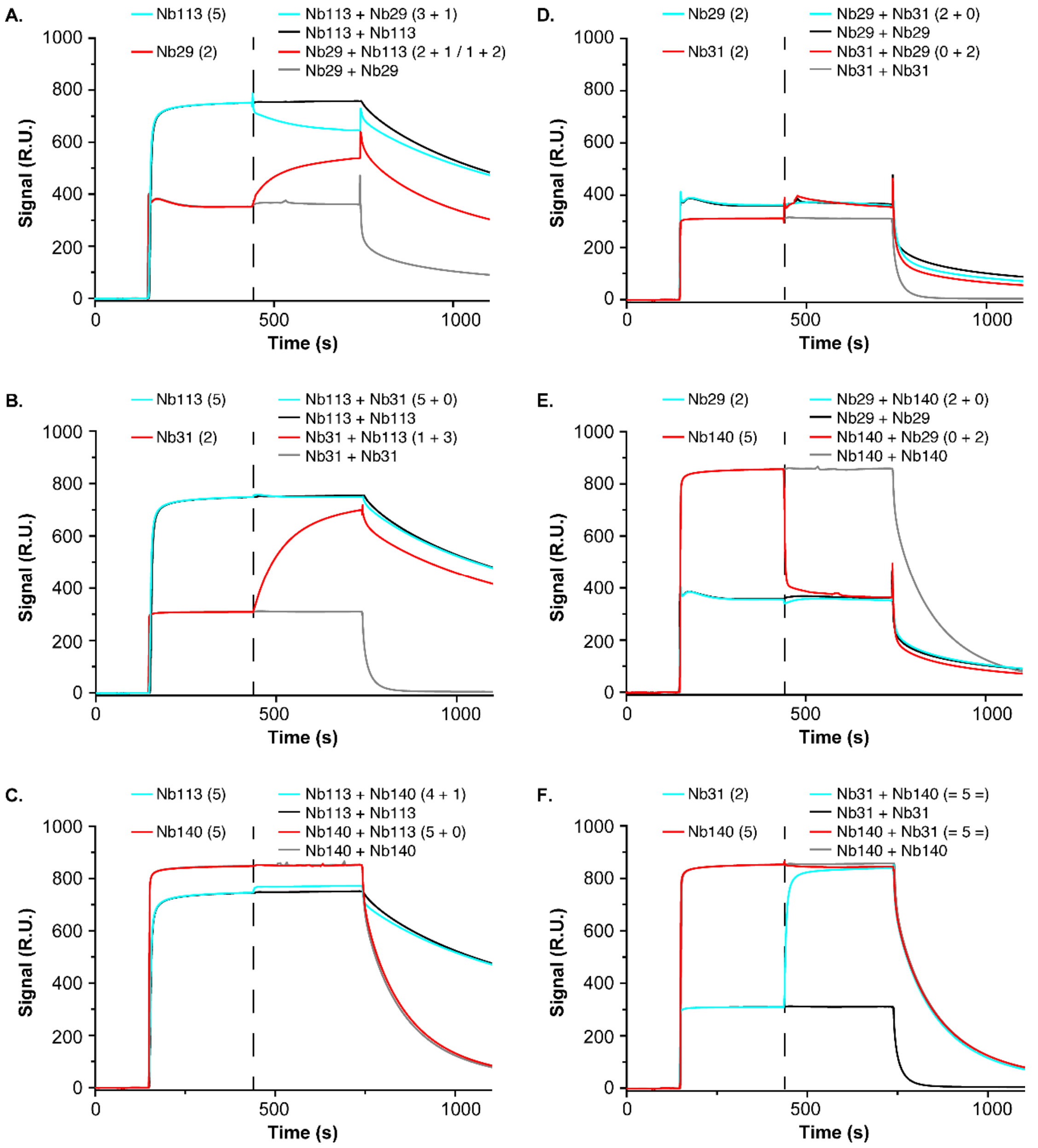

Figure S5. Epitope binning. A first nanobody (indicated left to the dashed line) was injected for 300 s at a concentration of 100x KD. From the time indicated by the dashed line we injected for 300 s a mixture of the first nanobody and a second nanobody (as indicated right to the dashed line). The second nanobody was also at a concentration of 100x KD. The number of nanobody molecules per pentameric rSt2xaB is indicated between brackets. (=5=) means that there are 5 nanobodies per pentamer rStx2aB but without knowing how much nanobody A and nanobody B are involved. In each panel we assessed the epitopes of two nanobodies. Nanobody 41 is not shown as it gives exactly the same sensorgrams as Nb140 since these two nanobodies belong to the same family and are binding to exactly the same epitope. In (**A**) we tested the Nb pair Nb29 and Nb113; (**B**) is Nb pair Nb31 and Nb113; (**C**) is Nb pair Nb113 and Nb140; (**D**) are the sensorgrams for Nb 29 and Nb 31; (**E**) shows the binning of Nb29 and Nb 140; (**F**) is the epitope mapping with Nb31 and Nb140.

Table S1. Main biochemical properties of rStx2aB-specific nanobodies and derivatives as calculated from Expasy ProtParam.

	**Vector**	**# AA**	**MW**	**pI**	**Ext coef.**
Nb29	pMECS	143	15375.04	7.88	23170
Nb31	pMECS	132	14519.03	8.00	24535
Nb41	pMECS	146	16183.75	8.96	31525
Nb140	pMECS	146	16135.69	8.61	30035
Nb113	pMECS	136	14735.21	7.98	31525
Nb113	pHEN6c	123	13364.77	9.01	27055
$Nb113_2$ [1]	pHEN6c	255	26834.53	9.14	54110
$Nb113_2$-SA1 [2]	pHEN6c	394	41445.55	8.98	61135

[1] Bivalent Nb construct. [2] Trimeric construct of bivalent $Nb113_2$ fused to Nb-SA1; #AA: number of amino acids

Table S2. Data collection and refinement statistics.

Data collection statistics	Nb113-rStx2aB
Wavelength (Å)	0.968610
Resolution range (Å)	47.47 - 3.00 (3.11 - 3.00)
Space group	hP:$P6_5$
Nb140	800
Nb113	700
Nb113$_2$ [1]	750
a,b,c (Å)	186.49,186.49,75.50
α,β,γ (°)	90,90,120
Mosaicity (°)	0.055
Total number of measured reflections	224155 (8342)
Unique reflections	30119 (2890)
Multiplicity	7.4 (2.9)
Completeness (%)	99.54 (96.62)
$\langle I/\sigma(I) \rangle$	9.31 (0.54)
Wilson B-factor (Å^2)	100.86
Rmeas (%)	16.75 (251.10)
CC1/2 (%)	99.50 (12.60)
A.U. contains	One Stx2aB pentamer bound by 5 Nb113 molecules
Refinement statistics	-
CC*	0.999 (0.474)
CC_{work}	0.914 (0.361)
CC_{free}	0.928 (0.349)
R_{work} (%)	19.08 (38.11)
R_{free} (%)	21.83 (38.84)
Number of non-hydrogen atoms	7128
macromolecules	7084
solvent	44
Protein residues	940
RMS bond lengths (Å)	0.015
RMS bond angles (°)	1.92
Ramachandran favored (%)	97.28
Ramachandran allowed (%)	2.72
Ramachandran outliers (%)	0.00
Rotamer outliers (%)	0.95
Clashscore	9.79
Overall MolProbity score	1.70
Average B-factor (Å^2)	119.53
macromolecules	119.71
solvent	91.03
PDB ID	6FE4

Statistics for the highest resolution shell are shown in parentheses.

Table S3. List of interactions between Nb113 and rStx2aB.

**Nb113**			**rStx2aB**			
Residue	Group	FR/CDR	Residue	Group	Interaction (distance)	# observations (out of 5)
Tyr33	side chain	CDR1	Trp48	side chain	hydrophobic	5
	side chain	CDR1	Ser50	side chain	Van der Waals	5
Trp47	backbone NH	FR2	Glu76	side chain	H-bond (2.99 ± 0.05 Å)	2
	backbone NH	FR2	Ser79	backbone CO	H-bond (2.82 Å)	1
	side chain	FR2	Trp48	side chain	hydrophobic	5
Asn52	side chain NH	CDR2	Asp35	backbone CO	H-bond (3.42 ± 0.57 Å)	5
Gly57	side chain	CDR2	Asp35	side chain	Van der Waals	5
Arg59	side chain	FR3	Asn33	side chain CO	H-bond (3.70 ± 0.44 Å)	5
	side chain	FR3	Asp35	side chain	electrostatic (3.20 ± 0.39 Å)	5
	side chain	FR3	Thr37	side chain OH	H-bond (2.97 ± 0.18 Å)	5
	side chain	FR3	Thr39	side chain OH	H-bond (3.81 ± 0.17 Å)	5
	side chain	FR3	Trp48	side chain	π stacking	5
Glu100	side chain	CDR3	Trp48	side chain	Van der Waals	5
	side chain	CDR3	Gly80	side chain	Van der Waals	5
	backbone CO	CDR3	Ser73	backbone NH	H-bond (4.11 ± 0.31 Å)	5
Gly102	backbone NH	CDR3	Gly80	backbone CO	H-bond (4.42 ± 0.16 Å)	5
	backbone CO	CDR3	Ser73	backbone NH	H-bond (2.92 ± 0.36 Å)	5
Asn103	side chain	CDR3	Glu34*	side chain	Van der Waals	5
Arg104	backbone NH	CDR3	Ser73	side chain	H-bond (3.68 ± 0.23 Å)	4
	side chain	CDR3	Ser73	side chain	H-bond (3.61 ± 0.33 Å)	4
	side chain	CDR3	Ser73	backbone CO	H-bond (4.26 ± 0.30 Å)	5
Tyr106	side chain	CDR3	Glu34*	side chain	Van der Waals	5

Distances only given in case of hydrogen bonds or electrostatic interaction. Also see Figure 3A.

Table S4. List of interactions between neighboring Nb113 molecules in the Nb113-rStx2aB complex.

**Nb113**			**Nb113**				
Residue	Group	FR/ CDR	Residue	Group	FR/ CDR	Interaction (distance)	# observations (out of 5)
Val2	side chain	FR1	Val56	side chain	CDR2	hydrophobic	5
Phe27	side chain	CDR1	Val56	side chain	CDR2	hydrophobic	5
Thr28	backbone NH	CDR1	Gly54	backbone CO	CDR2	H-bond (4.38 ± 1.14 Å)	5
Tyr32	side chain	CDR1	Val56	side chain	CDR2	hydrophobic	5
	side chain	CDR1	Asn52	side chain NH	CDR2	H-bond (4.83 ± 1.03 Å)	5
	side chain	CDR1	Val56	backbone NH	CDR2	H-bond (4.05 ± 0.77 Å)	5
	side chain	CDR1	Gly57	backbone NH	CDR2	H-bond (5.22 ± 0.94 Å)	5
Ile98	side chain	CDR3	Val56	side chain	CDR2	hydrophobic	5
Tyr106	side chain	CDR3	Val56	side chain	CDR2	hydrophobic	5
	side chain	CDR3	Thr58	backbone NH	CDR2	H-bond (4.37 ± 0.39 Å)	5

Distances only given in case of hydrogen bonds or electrostatic interaction. Also see Figure 3C. # stands for “number of”.
